# Trajectory-Based Skill Learning Using Generalized Cylinders

**DOI:** 10.3389/frobt.2018.00132

**Published:** 2018-12-18

**Authors:** S. Reza Ahmadzadeh, Sonia Chernova

**Affiliations:** ^1^Department of Computer Science, University of Massachusetts Lowell, Lowell, MA, United States; ^2^School of Interactive Computing, Georgia Institute of Technology, Atlanta, GA, United States

**Keywords:** learning from demonstration, trajectory-based skill, robot learning, physical human-robot interaction, skill refinement

## Abstract

In this article, we introduce Trajectory Learning using Generalized Cylinders (TLGC), a novel trajectory-based skill learning approach from human demonstrations. To model a demonstrated skill, TLGC uses a Generalized Cylinder—a geometric representation composed of an arbitrary space curve called the spine and a surface with smoothly varying cross-sections. Our approach is the first application of Generalized Cylinders to manipulation, and its geometric representation offers several key features: it identifies and extracts the implicit characteristics and boundaries of the skill by encoding the *demonstration space*, it supports for generation of multiple skill reproductions maintaining those characteristics, the constructed model can generalize the skill to unforeseen situations through trajectory editing techniques, our approach also allows for obstacle avoidance and interactive human refinement of the resulting model through kinesthetic correction. We validate our approach through a set of real-world experiments with both a Jaco 6-DOF and a Sawyer 7-DOF robotic arm.

## 1. Introduction

Learning from Demonstration (LfD) approaches provide the ability to interactively teach robots new skills, eliminating the need for manual programming of the desired behavior (Argall et al., 2009b). By observing a set of human-provided examples and constructing a model, LfD approaches can reproduce the skill and generalize it to novel situations autonomously. These capabilities make LfD a powerful approach that has the potential to enable even non-expert users to teach new skills to robots with minimum effort^1^. However, despite the existence of several trajectory-based skill learning approaches, the vast majority of the existing robotic platforms still rely on motion-level actions that are either hand-coded or captured through teleoperation by experts (Yanco et al., 2015), highlighting the need for further advances in this area. To be effective, trajectory-based learning representations should: (a) require few tuning parameters and be easy to tune especially by non-experts, (b) perform effectively and be robust to sub-optimal demonstrations, (c) generalize not only over the initial and final states but also to unforeseen situations successfully, and (d) support methods for refinement of the model constructed with the set of sub-optimal demonstrations.

While many LfD techniques exist that offer some subset of these requirements, no existing method fulfills all of these needs. In this paper, we present a novel LfD approach that meets all the above requirements through a geometric representation used to construct a model of the desired skill. The geometric representation is a generalized form of a standard cylinder, called a *Generalized Cylinder* (GC), for which the main axis is a regular curve in 3D Cartesian space (instead of a straight line) and the cross-sections can vary in size and shape (instead of circular cross-sections with fixed radius). We refer to the proposed approach as *Trajectory Learning using Generalized Cylinders (TLGC)*. One of the major advantages of employing generalized cylinders in our approach is that it allows for representing the *demonstration space* that implicitly encodes main characteristics of the demonstrated skill (i.e., the spatial correlations across different demonstrations).

In order to extract the underlying characteristics of the skill from the raw observations, TLGC requires minimal parameter tuning and can reproduce a variety of successful movements inside the boundaries of the encoded model by exploiting the whole demonstration space, thereby minimizing the effort of the user. Moreover, our representation is visually perceivable, which makes it a powerful candidate for physical human-robot interaction. This capability also helps to overcome the issue of sub-optimal demonstrations by enabling the user to improve the learned model through physical motion refinement. We show that unlike other existing techniques, using our approach refinements can be applied both through incremental and constraint-based strategies. Consequently, the user can start from a set of (sub-optimal) demonstrations and refine the learned model interactively to reach the desired behavior.

To tackle the problem of generalization over terminal states, we use a nonrigid registration method to transfer the encoded model accordingly. This generalization approach preserves the main characteristics of the demonstrated skill while achieving the goal of the task and satisfying a set of constraints. We also discuss an alternate generalization method that offers enhanced robustness. Additionally, TLGC offers several strategies for dealing with obstacles during the reproduction of the skill. In summary, our approach (a) maintains the important characteristics and implicit boundaries of the skill by encoding the demonstration space, (b) requires minimal parameter tuning, (c) reproduces a variety of successful movements by exploiting the whole demonstration space, (d) generalizes over the terminal states of the skill by deforming the model while preserving its important characteristics, (e) enables users to provide physical feedback to improve the characteristics/quality of the learned skill interactively, and (f) offers multiple obstacle avoidance strategies.

In our prior work (Ahmadzadeh et al., 2016), we encoded a set of demonstrations as a *Canal Surface* (CS), a simpler form of a generalized cylinder, and showed that multiple solutions of a skill can be reproduced inside the CS. We then considered a more flexible and generalized form for the representation with the use of generalized cylinders (Ahmadzadeh et al., 2017). In this article, we merge prior work and extend the idea by introducing (a) a novel reproduction strategy with more flexibility, (b) an alternate method for generalization of skills with more robustness, (c) evaluation and comparison of generalization methods, (d) additional comparisons of skill reproduction and refinement against other approaches, and (e) three obstacle avoidance strategies.

We validate our approach in fourteen experiments using two physical 6 and 7-DOF robots, as well as demonstrate its use in comparison to Dynamic Movement Primitives (Ijspeert et al., 2013), Gaussian Mixture Models (GMM) (Calinon et al., 2007), and GMM with weighted Expectation-Maximization (Argall et al., 2010).

## 2. Related Work

In this section, we review related work on LfD approaches that are designed for modeling and reproduction of trajectory-based skills (i.e., movements). LfD approaches differ in the way they encode a demonstrated skill and retrieve a generalized form of the skill (Argall et al., 2009b). One category of approaches use probabilistic representations generated through regression (Vijayakumar et al., 2005; Grimes et al., 2006; Calinon et al., 2007). Work by Calinon et al. (2007) uses a Gaussian Mixture Model (GMM) and retrieves a smooth trajectory using Gaussian Mixture Regression (GMR). The reproduced trajectory using GMR is attracted toward an average form of the demonstrated skill and cannot adapt to changes in initial and final states. To improve its generalization capabilities, a task parameterized extension of GMM/GMR was developed that assigns reference frames to task-related objects and landmarks (Calinon, 2016). The resulting method generalizes better to novel situations but requires extensive parameter tuning for each trajectory (e.g., number of Gaussian components, scale, weight, kernel). Our approach generalizes to novel situations without the use of reference frames and requires minimal parameter tuning.

Grimes et al. (2006) employed Gaussian Process regression to learn and generalize over a set of demonstrated trajectories. Although Gaussian Processes (GPs) provide a non-parametric alternative, the computational complexity of conventional GP approaches scales cubically with the number of data points, limiting their effectiveness in trajectory-based LfD settings. To address this issue, in follow-on work, Schneider and Ertel (2010) used local Gaussian process regression. Another approach called LfD by Averaging Trajectories (LAT) used only one-dimensional normal distributions to reach lower computational cost (Reiner et al., 2014). Both GPs and LAT reproduce an average form of the movement and cannot generalize to novel situations (e.g., terminal states). Our approach can reproduce multiple successful solutions of the demonstrated skill and generalize according to the changes in the terminal states as well as in the environment.

An alternative to probabilistic approaches is to use dynamic systems to encode and reproduce skills (Hersch et al., 2006; Khansari-Zadeh and Billard, 2011; Ijspeert et al., 2013). Dynamic Movement Primitives (DMPs) represent demonstrations as movements of a particle subject to a set of damped linear spring systems perturbed by an external force (Ijspeert et al., 2013). The shape of the movement is approximated using Gaussian basis functions and the weights are calculated using locally weighted regression. DMPs can handle generalization of the skill to new goal situations, however, the implicit definition of time as a canonical system the movement becomes slower as time increases. The implicit time dependency also makes the system sensitive to temporal perturbations. Finally, to maintain the shape of the movement during generalization, DMPs require significant tuning of continuous parameters, including those of the dynamic systems, such as time constants and scaling factors. Unlike DMPs, our approach is time-independent, requires minimal parameter tuning, and reproduces trajectories that do not converge to an average solution.

Khansari-Zadeh and Billard (2011) introduced the Stable Estimator of Dynamical Systems (SEDS) approach which uses a constrained optimization technique to model a set of demonstrations as a dynamic system. Unlike DMPs, SEDS is robust to perturbations and ensures global asymptotic stability at the target. However, it requires a goal state and fails if the demonstrations do not converge to a single final state. It also cannot handle via-points (i.e., points where all demonstrations pass through with a very small variance). Similar to DMPs, SEDS relies on the first derivative of the motion (i.e., velocity) whether it is given through demonstrations or computed internally. Our approach does not require velocity data, can learn to move through via-points, and can handle demonstrations with different final states.

Other approaches such as Probabilistic Movement Primitives (ProMP) (Paraschos et al., 2013) and Combined Learning from demonstration and Motion Planning (CLAMP) (Rana et al., 2017) approximate a stochastic control policy in the form of a Gaussian Process. ProMP directly fits a Gaussian distribution into demonstrations and then finds a control policy to reproduce the skill and satisfy the constraints from the skill and the environment. CLAMP, on the other hand, generates trajectories that naturally follow the demonstrated policy while satisfying the constraints. These approaches can reproduce various solutions within the Gaussian distribution, however, both are limited in modeling the movement as a discrete-time linear dynamic system.

Several other techniques utilize models with characteristics similar to generalized cylinders (Quinlan and Khatib, 1993; Majumdar and Tedrake, 2016). Quinlan and Khatib (1993) proposed elastic bands to tackle the real-time obstacle avoidance in motion planning. Similar to our representation, their approach assigns a set of bubbles (i.e., 2D circles) to a global solution. By applying small and local changes to the constructed model the global path can be deformed. However, the approach is limited to planar applications. The real-time motion planning approach proposed by Majumdar and Tedrake (2016) approximates a boundary around a trajectory (similar to elastic bands), which is visualized as a funnel. The generated funnels illustrate a similar representation to our approach, however, TLGC does not require extensive off-line computation. Dong and Williams (2012) proposed probabilistic flow tubes to represent trajectories by extracting covariance data. The learned flow tube consists of a spine trajectory and 2D covariance data at each corresponding time-step. Although the approach was applied to extract a human's intention, the flow tube representation can be seen as a special case of TLGC in which the cross-sections are formed using the covariance data. Generalized cylinders have been used in the context of providing safe human-robot interaction (Mart́ınez-Salvador et al., 2003; Corrales et al., 2011). To avoid collisions between a robot arm and a human, Mart́ınez-Salvador et al. (2003) used GCs to build a spatial model and proposed a computationally effective method for collision checking. In the approach proposed by Corrales et al. (2011), however, the shape of the GCs representing the robot also changes according to the robot's speed.

Regardless of the technique used for learning from demonstration, the capability of improving the learned model by refining its shape or spatial constraints is highly desirable. This can become available through physical human-robot interaction. There exist few approaches that enable the human to refine the initially given demonstrations. Argall et al. (2010) used tactile feedback for refining a given set of demonstrations and reusing the modified demonstrations to reproduce the skill through incremental learning. They used this approach for performing grasp-positioning tasks on a humanoid robot. Lee and Ott (2011) also proposed an incremental learning approach for iterative motion refinement. Their approach combines kinesthetic teaching with impedance control and represents the skill using a Hidden Markov Model (HMM). Our approach, on the other hand, can be used to refine the learned skill by applying user-provided corrections for both demonstrations and reproductions interactively.

## 3. Background

Consider a smooth simple closed-curve *ρ*, which is a non-self-intersecting continuous loop^2^ in the plane ℝ^2^, perpendicular to an arbitrary regular curve^3^ Γ in Cartesian space ℝ^3^ (see Figure 2A). A *Generalized Cylinder* (GC) is a 3D surface generated by translating the plane containing the closed-curve *ρ* along the arbitrary curve Γ while keeping the plane perpendicular to Γ at each step. We refer to Γ and *ρ* as the *directrix* (also spine) and the *cross-sectional curve*, respectively. While translating along the directrix, the cross-sectional curve can vary smoothly in both shape and size. Figure 2B illustrates six GCs with identical directrices but different cross-sectional functions.

**Figure 1:**
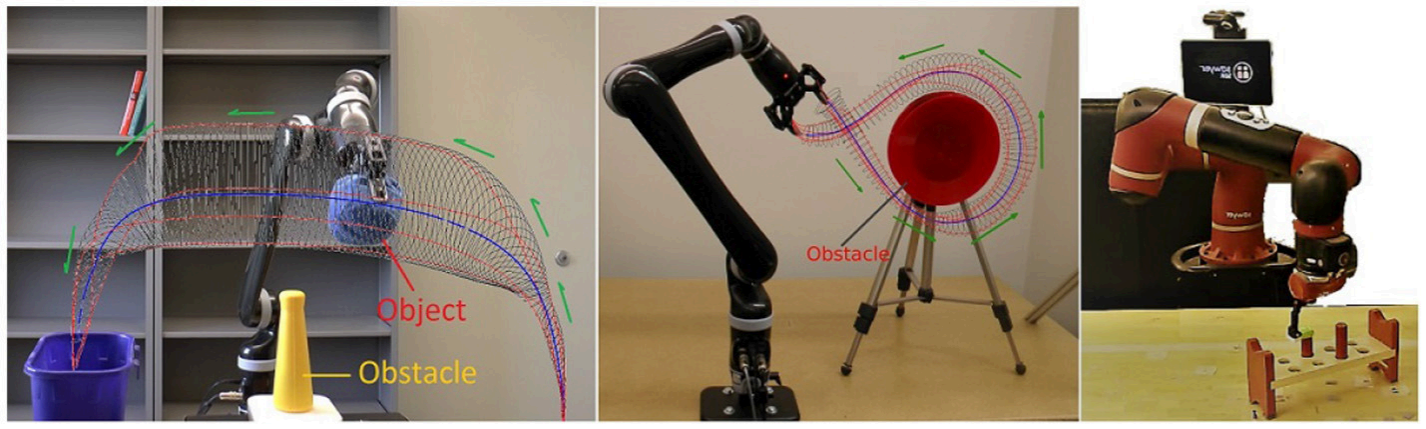
Two robots reproducing three trajectory-based skills encoded and learned using TLGC.

**Figure 2:**
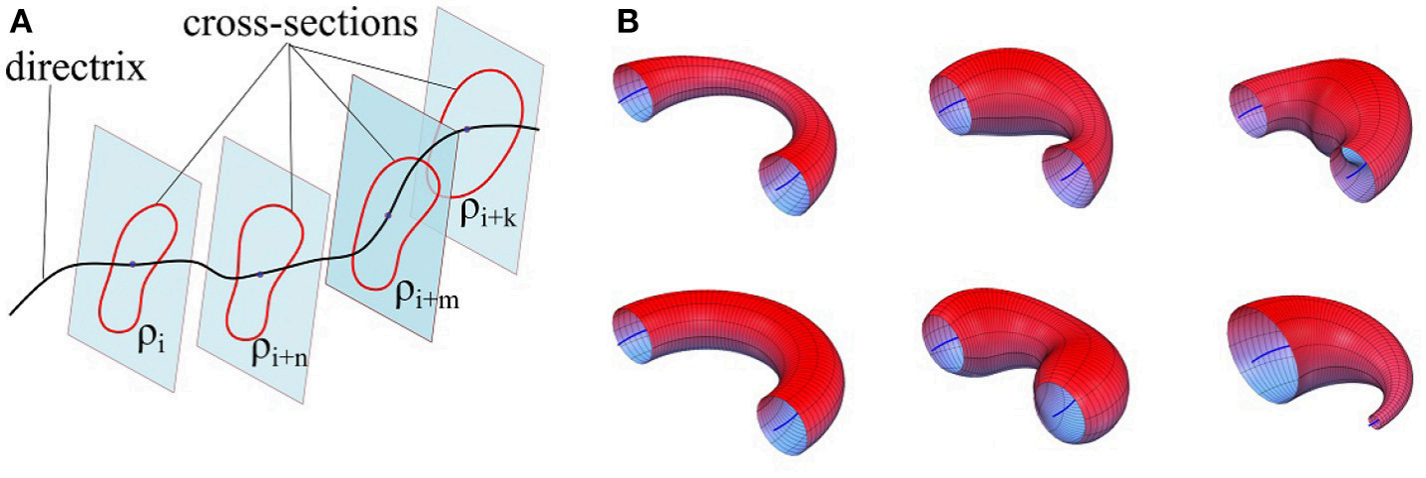
**(A)** Formation of a generalized cylinder by translating the cross-sectional curve along the directrix. **(B)** Six generalized cylinders with identical directrices and different cross-sectional functions.

Generalized cylinders play a fundamental role in Differential Geometry, and in the context of Computer Aided Graphic Design, their application includes construction of smooth blending surfaces, shape reconstruction, and transition surfaces between pipes (Hartmann, 2003). In robotics, generalized cylinders have been used for finding flyable paths for unmanned aerial vehicles (Shanmugavel et al., 2007). They also have been used for collision detection during physical human-robot interaction (Mart́ınez-Salvador et al., 2003; Corrales et al., 2011). To our knowledge, this is the first application of generalized cylinders to skill learning for manipulation. In this section, we first outline the mathematical definition and parameterized formulation of *Canal Surfaces* (CS) (Hilbert and Cohn-Vossen, 1952), which are a simpler form of GCs, and then extend the formulae to generalized cylinders.

### 3.1. Canal Surfaces

Let ℝ^3^ be Euclidean 3-space with Cartesian coordinates *x*_1_, *x*_2_, *x*_3_. Let Φ_*u*_ be the one-parameter pencil^4^ of regular implicit surfaces^5^ with real-valued parameter *u*. Two surfaces corresponding to different values of *u* intersect in a common curve. The surface generated by varying *u* is the envelope^6^ of the given pencil of surfaces (Abbena et al., 2006). The envelope can be defined by

(1) $$\begin{array}{r} {\text{Φ}}_{u}:F\left(\text{x};u\right)=0, \end{array}$$

(2) $$\begin{array}{r} \frac{\partial F\left(\text{x};u\right)}{\partial u}=0, \end{array}$$

where $\text{x}={\left[x_{1},x_{2},x_{3}\right]}^{⊤}$ and Φ_*u*_ consists of implicit *C*^2^−surfaces which are at least twice continuously differentiable. A **canal surface** $C_{u}$ is defined as an envelope of the one-parameter pencil of spheres and can be written as

(3) $$\begin{array}{r} C_{u}:f\left(\text{x};u\right):=\left\{\left(\text{c}\left(u\right),r\left(u\right)\right)\in {\mathbb{R}}^{3,1}|u\in \mathbb{R}\right\}, \end{array}$$

where the spheres are centered on the regular curve Γ: **x** =**c**(*u*) ∈ ℝ^3^ in Cartesian space. The radius of the spheres are given by the function *r*(*u*) ∈ ℝ, which is a *C*^1^-function. The non-degeneracy condition is satisfied by assuming *r* > 0 and $\left|ṙ|<||\overset{{}^{\circ}}{\text{c}}|\right|$ (Hartmann, 2003). Γ is the *directrix* (spine) and *r*(*u*) is the cross-sectional function which in this case is called the *radii* function. For the one-parameter pencil of **spheres**, Equation (3) can be written as

(4) $$\begin{array}{r} C_{u}:f\left(\text{x};u\right):={\left(\text{x}-\text{c}\left(u\right)\right)}^{2}-r{\left(u\right)}^{2}=0. \end{array}$$

Two canal surfaces with fixed and varying cross-sections have been depicted in Figure 2B (bottom-left) and Figure 2B (top-left) respectively.

### 3.2. Generalized Cylinders

Since canal surfaces are constructed using the one-parameter pencil of spheres, the cross-sectional curve is always a circle even though its radius can vary along the directrix. Generalized cylinders extrapolate this notion by considering an arbitrary cross-sectional curve that can vary in both shape and size while sweeping along the directrix. These variations make generalized cylinders a powerful candidate for modeling complicated constraints of trajectory-based skills captured through demonstrations. We define a **generalized cylinder** $G_{u,v}$ as

(5) $$\begin{array}{r} G_{u,v}:f\left(\text{x};u,v\right):=\left\{\text{c}\left(u\right),\rho \left(u,v\right)\in {\mathbb{R}}^{3,2}|u,v\in \mathbb{R}\right\}, \end{array}$$

where *ρ*(*u, v*) represents the cross-sectional function with parameters *u*, the arc-length on the directrix, and *v*, the arc-length on the cross-sectional curve. The dependence on *u* reflects the fact that the cross-section's shape may vary along the directrix. To obtain a parametric representation for generalized cylinders, it is useful to employ a local coordinate system defined with origin on the directrix. A convenient choice is the Frenet-Serret (or TNB) frame which is suitable for describing the kinematic properties of a particle moving along a continuous and differentiable curve in ℝ^3^. TNB is an orthonormal basis composed of three unit vectors: the unit tangent vector **e**_*T*_, the unit normal vector **e**_*N*_, and the unit binormal vector **e**_*B*_. For a non-degenerate directrix curve Γ:**x**(*u*), the TNB frame can be defined by

(6) $$\begin{array}{ll} e_{T}(u) & =\frac{dx(u)}{du},\\ e_{N}(u) & =\frac{de_{T}}{du}/\|\frac{de_{T}}{du}\|,\\ e_{B}(u) & =e_{T}\times e_{N}, \end{array}$$

where ||.|| denotes the Euclidean norm of a vector and × denotes the cross product operation. Since **e**_*T*_ is tangent to the directrix, we keep the cross-sectional curve in the plane defined by **e**_*N*_ and **e**_*B*_. Using this convention, we form a parametric representation of generalized cylinders as

(7) $$\begin{array}{r} G_{u,v}:f\left(u,v\right):=\text{c}\left(u\right)+\rho_{x_{1}}\left(u,v\right){\text{e}}_{N}\left(u\right)+\rho_{x_{2}}\left(u,v\right){\text{e}}_{B}\left(u\right). \end{array}$$

Calculating Frenet-Serret frames for real data is prone to noise. The reason is that at some points the derivative vector $\frac{d{\text{e}}_{T}}{du}$ vanishes and the formulae cannot be applied anymore (i.e., **e**_*N*_ becomes undefined at the points where the curvature is zero). This problem can be addressed by calculating the unit normal vector **e**_*N*_ as the cross product of a random vector by the unit tangent vector **e**_*T*_. In this article, we use an improved technique called Bishop frames (Bishop, 1975) which tackles the issue by employing the concept of relatively parallel fields. Alternate techniques, such as Beta frames (Carroll et al., 2013) can also be employed.

## 4. Skill Learning Using Generalized Cylinders

**Figure 3:**
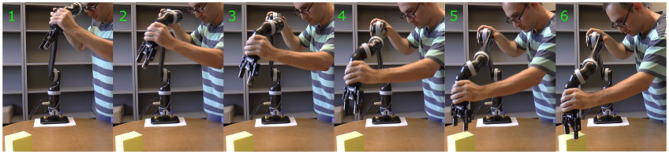
One of the authors of the paper demonstrating a reaching skill using kinesthetic teaching. Captured task-space poses of the end-effector are used as input for learning.

In this section, we explain how generalized cylinders can be used to encode, reproduce, and generalize trajectory-based skills from demonstrations. We assume that multiple examples of a skill are demonstrated and captured as a set of trajectories. To capture demonstrations we use kinesthetic teaching (Figure 3), however, alternate demonstration techniques, such as teleoperation and shadowing, can be employed (Argall et al., 2009b). Given the set of captured demonstrations, our approach first calculates the directrix (i.e., an average form of the movements) and then extracts the main characteristics of the set (i.e., spatial correlations across demonstrations) and forms the cross-sectional function by identifying its boundaries.

When the GC is constructed, a geometric approach is used for generating new trajectories starting from arbitrary initial poses. We also estimate a transformation function that generalizes the encoded skill over terminal constraints (i.e., novel initial and final poses). Algorithm 1 shows a pseudo code of the proposed approach.

**Table:** **Algorithm 1** Skill Learning using Generalized Cylinders

1:	procedure Encoding demonstrations
2:	Input:set of *n* demonstrations **ξ**∈ℝ^3 × *N*×*n*^
3:	Output:Generalized cylinder $G_{u,v}$, TNB frames F(u)
4:	**m**(*u*)←*mean*(**ξ**)
5:	P(u,v)←estimateCSpline(ξ)
6:	$G_{u,v},F\leftarrow \text{makeGeneralizedCylinder}\left(\text{m}\left(u\right),P\left(u,v\right)\right)$
7:	**procedure**REPRODUCING TRAJECTORY
8:	Input:initial point ${\text{p}}_{0}\in {\mathbb{R}}^{3}$, $G_{u,v}$, F
9:	Output:New trajectory **ρ**∈ℝ^3 × *N*^
10:	$\eta \leftarrow \frac{\left||{\text{p}}_{0}-{\text{c}}_{0}|\right|}{\left||{\text{g}}_{0}-{\text{c}}_{0}|\right|}$
11:	**p**_*i*_←**p**_0_ , **ρ**←**p**_0_
12:	for each $\text{frame}F_{i}$**do**
13:	${\text{p}}_{i+1}\leftarrow \text{project}\left({\text{p}}_{i},\eta ,F_{i+1},F_{i}\right)$
14:	**ρ**←**p**_*i*+1_
15:	*i*←*i*+1

### 4.1. Skill Encoding

Consider *n* different demonstrations of a task performed and captured in task-space. For each demonstration, the 3D Cartesian position of the target (i.e., robot's end-effector) is recorded over time as ${\hat{\xi }}^{j}=\{\xi_{1}^{j},\xi_{2}^{j},\xi_{3}^{j}\}⊤\in {\mathbb{R}}^{3\times T^{j}}$, *j* = 1, …, *n*, where *T*^*j*^ is the number of data-points within the *j*th demonstrated trajectory. Since *T*^*j*^ can vary among demonstrations, we use interpolation and resampling in order to gain a frame-by-frame correspondence mapping among the recorded demonstrations and align them temporally. To achieve this, for each demonstration, we obtain a set of piecewise polynomials using cubic spline interpolation. Then, we generate a set of temporally aligned trajectories by resampling *N* new data-points from each obtained polynomial. This process results in a set of *n* resampled demonstrations **ξ** ∈ ℝ^3 × *N*×*n*^ each of which consists of *N* data-points. An advantage of this technique is that when the velocity and acceleration data are unavailable, the first and second derivatives of the estimated polynomials can be used instead. An alternate solution is to employ Dynamic Time Warping (Myers et al., 1980).

#### 4.1.1. Estimating the Directrix

To estimate the directrix Γ, we calculate the directional mean (axis-wise arithmetic mean) for the set of demonstrations. Let **m** ∈ ℝ^3 × *N*^ be the directional mean of set **ξ** (Line 4 in Algorithm 1). Note that all the cross-sections will be centered on **m**. Alternatively, the directrix can be produced using Gaussian Mixture Regression (GMR) (Calinon et al., 2007). In that case, GMR generates the directrix by sampling from the joint probability learned by GMM. However, using GMR requires an explicitly defined time vector.

**Table:** **Algorithm 2** Generating GC with arbitrary cross-section

1:	procedure makeGeneralizedCylinder
2:	Input:directrix **m**(*u*), boundary function P(u,v)
3:	Output:Generalized cylinder $G_{u,v}$, TNB frames $F_{u}$
4:	for each *u*_*i*_**do**
5:	{**e**_*T*_(*u*_*i*_), **e**_*N*_(*u*_*i*_), **e**_*B*_(*u*_*i*_)}←*estimateFrame*(**m**(*u*_*i*_))
6:	$F\leftarrow \left\{{\text{e}}_{T}\left(u_{i}\right),{\text{e}}_{N}\left(u_{i}\right),{\text{e}}_{B}\left(u_{i}\right)\right\}$
7:	$G_{u,v}\leftarrow \text{m}\left(u_{i}\right)+P^{x}\left(u_{i},v\right){\text{e}}_{N}\left(u_{i}\right)+P^{y}\left(u_{i},v\right){\text{e}}_{B}\left(u_{i}\right)$

#### 4.1.2. Estimating the Cross-Section Function

**Figure 4:**
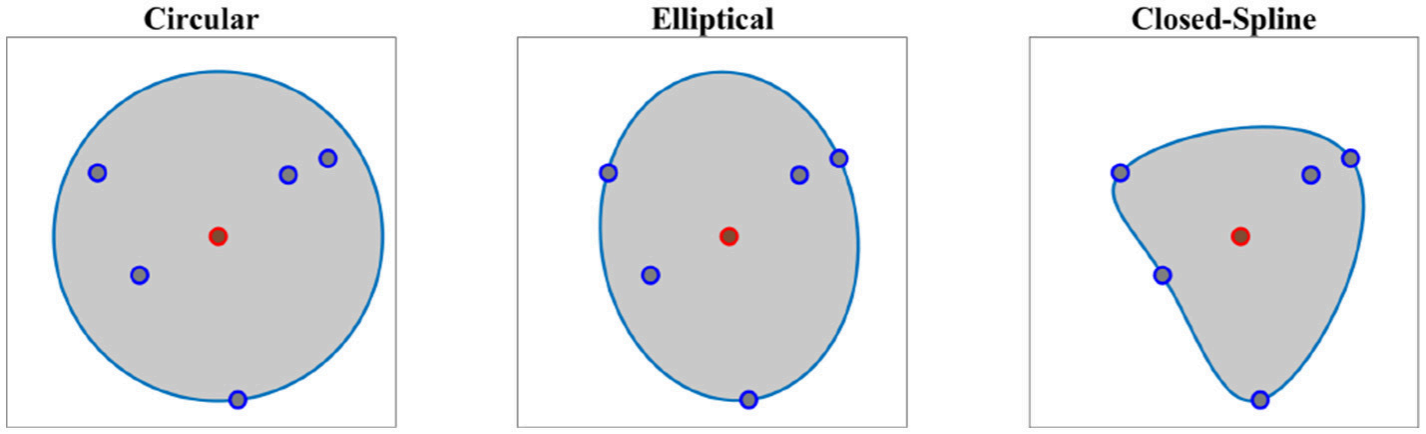
Different types of cross-section on the same set of data. Point on the directrix and the effective points are shown in red and blue respectively.

**Figure 5:**
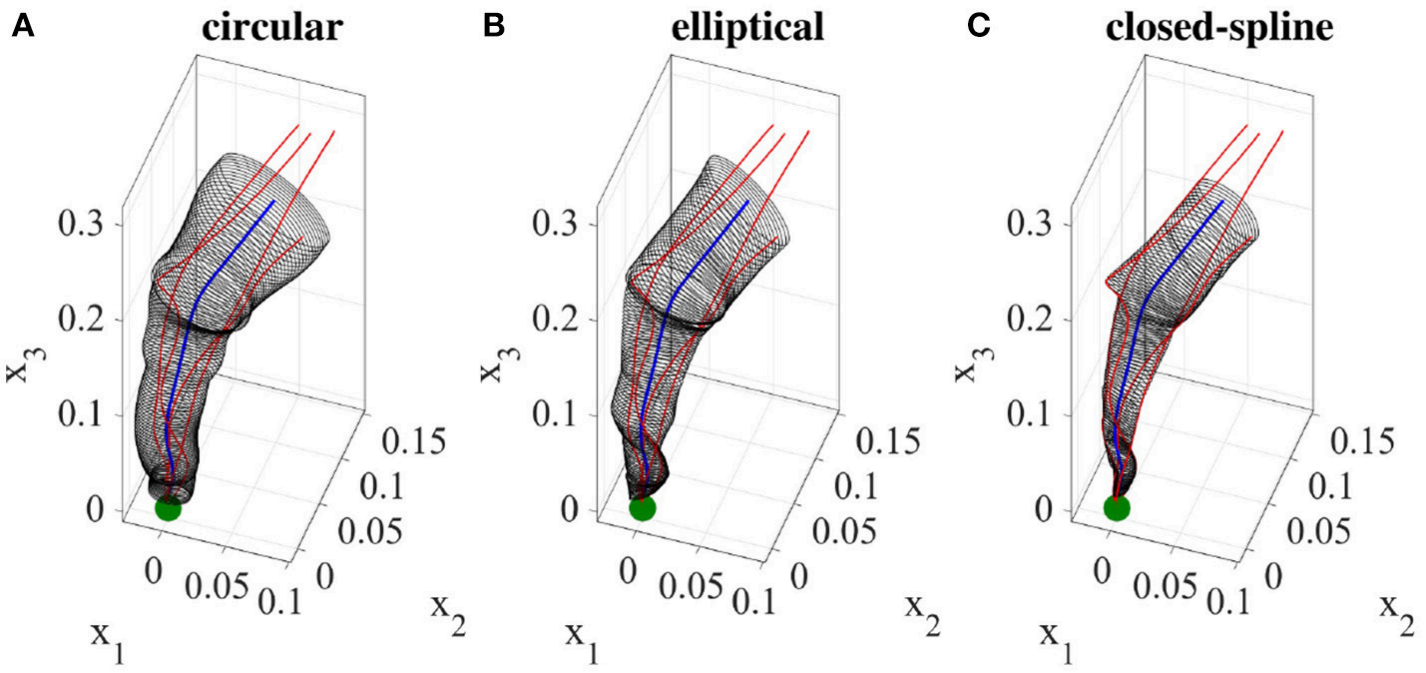
A reaching skill encoded using three generalized cylinders with different cross-section types. The demonstrations and the object are shown in red and green respectively.

Given the demonstration set **ξ** and the estimated directrix **m**, in this step, we explain methods for calculating the cross-sectional function *ρ*(*u, v*). For each point *u* on the directrix, we gather one corresponding point (aligned with *u* on the same cross-sectional plane) from each demonstration; we call this set the *effective points*. As an example, for a set of five demonstrations, one point on the directrix and the corresponding effective points are depicted in Figure 4 in blue and red respectively. We use the effective points to calculate the cross-section at each step with a smooth closed curve. The circumference of a cross-section represents the implicit local constraints of the task (i.e., boundaries) imposed by the set of demonstrations. Figure 4 illustrates three different types of cross-sections calculated for the same set of effective points. In its simplest form, we can employ (4) and construct a canal surface which has a circular cross-section. The radius of each circle is equal to the distance from the point on the directrix to the furthest effective point (i.e., point with maximum distance). As shown in Figure 4 (left), the estimated cross-section bounds the other effective points as well and consequently the formed canal surface encloses all the demonstrations. The radii function *r*(*u*) ∈ ℝ of the obtained canal surface assigns a radius for each point *u*. We use a constant range *v* to parameterize the circumference of the circular cross-section (e.g., *v* = [02π]). More detail on encoding skills using canal surfaces can be found in Ahmadzadeh et al. (2016). To cover the cross-sectional area more effectively and precisely while maintaining the implicit local constraints of the task, we can also construct generalized cylinders with elliptical cross-sections [see Figure 4 (middle)]. The radii function for elliptical cross-section **r**(*u*):ℝ↦ℝ^3^ produces the major and minor axes and the rotation angle of the ellipse at each step *u*. In a more general form, we generate cross-sections by interpolating closed splines to the data. Given a set of break points *v*_*j*_, *j* = 1, …, *m* on the interval [*v*_0_, *v*_*m*_] such that *v*_0_<*v*_1_ < … < *v*_*m*−1_<*v*_*m*_, we can fit a cubic polynomial $p\left(v\right)=a_{0}+a_{1}\left(v-v_{0}\right)+a_{2}{\left(v-v_{0}\right)}^{2}+a_{3}{\left(v-v_{0}\right)}^{3}$ to each interval described with four coefficients *a*_0_, *a*_1_, *a*_2_, *a*_3_. The accumulated square root of chord length is used to find the breaks and the number of polynomials. Since each polynomial is *C*^2^-continuous, by applying the boundary condition $p^{\prime \prime }\left(t_{0}\right)=p^{\prime \prime }\left(t_{n}\right)=0$ and joining the polynomials, we construct a smooth piecewise polynomial curve called a closed cubic spline. The obtained closed-spline denoted by P(u,v) is *C*^2^-continuous within each interval and at each interpolating nodes. Figure 4 (right) shows a closed-spline cross-section constructed on the same set of effective points. Figure 5 depicts three GCs with circular, elliptical and closed-spline cross-sections constructed for a reaching skill toward an object (the green sphere).

Finally, as mentioned earlier, the presented approach requires minimal parameter tuning in that only the shape of the cross-section needs to be defined. However, we have found the closed-spline cross-section to be most effective in encoding a wide range of trajectories, thus serving as a useful default for this single parameter.

### 4.2. Skill Reproduction

**Figure 6:**
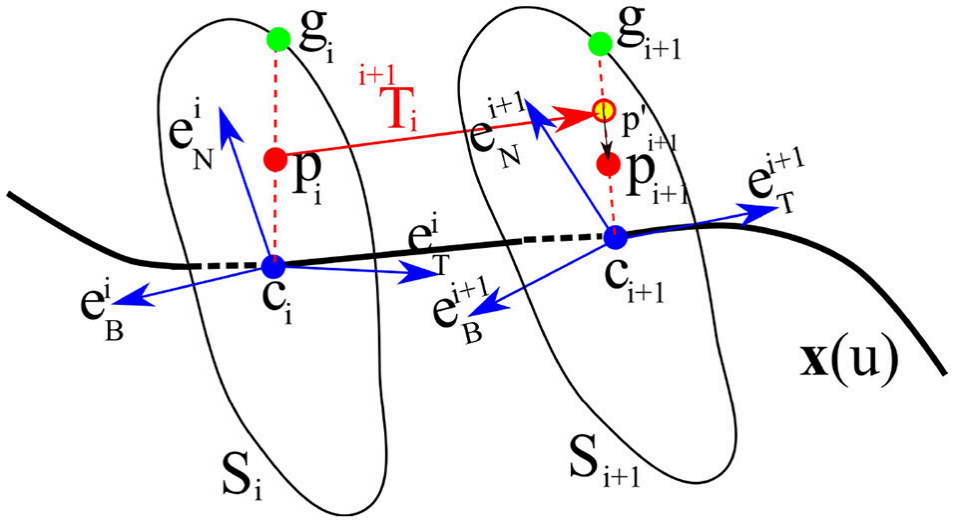
Reproduction from a random initial pose *p*_*i*_ on the *i*th cross-section *S*_*i*_ to the next cross-section *S*_*i*+1_ using projection ${\overset{´}{p}}_{i+1}{=}^{F_{i+1}}T_{F_{i}}p_{i}$ and scaling $p_{i+1}=\left(\eta .||\bar{g_{i+1}c_{i+1}}||\right){\overset{´}{p}}_{i+1}$.

During the reproduction phase, the initial position of the end-effector *p*_0_ in the cross-sectional plane *S*_0_ (perpendicular to the directrix at *c*_0_) is used as input. This point can be either provided by the current pose of the robot end-effector or generated randomly. By drawing a ray starting from *c*_0_ and passing through *p*_0_, we find *g*_0_, the intersection of the ray and the cross-sectional curve (see Figure 6, for *i* = 0). We consider the distance between the given point *p*_0_ to *g*_0_ as a measure of the similarity of the movement we want to reproduce to the nearest neighbor on the GC. We define this similarity by measuring the ratio η (Line 10 in Algorithm 1) by

(8) $$\eta =\frac{\left\|\bar{p_{0}c_{0}}\right\|}{\left\|\bar{g_{0}c_{0}}\right\|}.$$

To calculate the next point of the trajectory, we first transform *p*_0_ from the current TNB frame $F_{0}$ to the next frame $F_{1}$ using ${\overset{´}{p}}_{1}{=}^{F_{1}}T_{F_{0}}p_{0}$ and then similar to the previous step, we find *g*_1_, the intersection of the ray started from *c*_1_ passing through ${\overset{´}{p}}_{1}$. We then use η to adjust the projected point ${\overset{´}{p}}_{1}$ according to the measured ratio and the size and shape of the cross-section *S*_1_ by

(9) $$p_{1}=(\eta \|\bar{g_{1}c_{1}}\|){\overset{´}{p}}_{1}.$$

We then repeat this process for each cross-section. Since throughout the process, the ratio η is kept fixed, we call this reproduction method the *fixed-ratio rule*. An illustration of a single-step reproduction process using the fixed-ratio rule can be seen in Figure 6. Using this method we can generate new trajectories from any point inside the generalized cylinder (i.e., within the demonstration space) and ensure that the essential characteristics of the demonstrated skill are preserved. Another advantage of this reproduction strategy is in its high computational efficiency since calculating each point requires a projection followed by a scaling. A demo implementation of TLGC with the fixed-ratio reproduction strategy is available online^7^.

#### 4.2.1. Adaptive-Ratio Strategy

**Figure 7:**
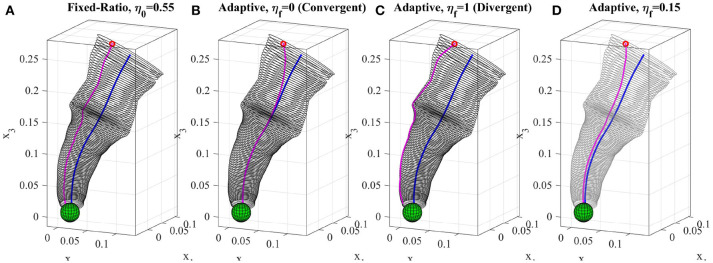
Reproduction of the reaching skill using different reproduction strategies (section 4.2). **(A)** fixed-ratio rule with η_0_ = 0.55, **(B)** adaptive-ratio rule with η_*f*_ = 0 (i.e., convergent), **(C)** adaptive-ratio rule with η_*f*_ = 1 (i.e., divergent), **(D)** adaptive-ratio rule (in dark green) with η_0_ = 0.55 and η_0_ = 15. The directrix and the reproductions are depicted in blue and magenta respectively. All trajectories were reproduced from the same initial point.

While keeping a fixed ratio enables the robot to reproduce the skill while preserving the important characteristics of the movement (i.e., shape), being able to change the ratio from its initial value to a specific value introduces new capabilities. We call this procedure the adaptive-ratio strategy and later in section 7.2, we show the effectiveness of this strategy for handling obstacles. In this strategy, the transition from the initial ratio η_0_ to a target ratio η_*f*_ should be done smoothly. Although different methods can be used to achieve this goal, we utilize the exponential decay given by

(10) $$\begin{array}{r} \eta_{i+1}=\left(\eta_{0}-\eta_{f}\right)e^{-\gamma u_{i}}+\eta_{f}, \end{array}$$

where η_*i*+1_ is the ratio at step *u*_*i*_ and γ denotes the decay constant. Using (10) together with (8) and (9), the ratio smoothly converges from its initial value η_0_ to the desired value η_*f*_. Figure 7A shows one reproduction of the reaching skill from a given initial point using the fixed-ratio of η_0_ = 0.55. The reproduced trajectory preserves its shape and remains with similar distance from the directrix. Whereas, Figure 7D shows the reproduction of the reaching skill from the same initial point using the adaptive-ratio strategy with η_0_ = 0.55 and η_*f*_ = 0.15. It can be seen that the reproduced trajectory converges toward the directrix but keeps a distance according to the final ratio value. In section 7.2, we provide a method for estimating η_*f*_ and γ automatically from a detected obstacle. In the rest of this section, we discuss two cases where selection of η_*f*_ produces two different reproduction behaviors.

#### Convergent Strategy

In most LfD approaches, the reproduction always converges toward the mean of the learned model regardless of the location of the initial point (for example see results produced using DMPs Calinon et al., 2010 in Figure 11). This behavior is suitable when for instance the covariance information of the learned model represent the uncertainty and a reproduced trajectory should avoid staying in uncertain areas and converge toward the mean which is considered to be the most certain shape of the skill learned from the demonstrations. We show that TLGC can mimic such behavior by decaying the initial ratio exponentially from η_0_ to η_*f*_ = 0. In this case, Equation (10) can be written as $\eta_{i+1}=\eta_{0}e^{-\gamma u_{i}}$. Using this formula together with (8) and (9), the ratio smoothly converges to zero while consequently the reproduced trajectory gradually converges toward the directrix. Figure 7B depicts the reproduction of the reaching skill using the convergent strategy (i.e., η_0_ = 0.55 and η_*f*_ = 0). This experiment shows that TLGC can reproduce trajectories similar to the ones reproduced by Dynamic Movement Primitives (DMPs) (Ijspeert et al., 2013).

#### Divergent Strategy

While using the convergent strategy the reproduction mimics the behavior of the directrix, we can think of a case where we want the trajectory to remain on the boundary of the generalized cylinder. Such reproductions use the limits of the demonstration space provided by the human teacher. This can be seen as another method for avoiding uncertain areas. Since the provided demonstrations by the teacher do not include any information about the area they enclose, reproducing a trajectory similar to the directrix might not always be desirable or even safe. In other words, the user might prefer to stay as close as possible to the the known areas of the demonstration space and reproduce the skill similar to the nearest observed examples. Such behavior can be achieved by decaying the initial ratio exponentially from η_0_ to η_*f*_ = 1. Equation (10) can be simplified to $\eta_{i+1}=\left(\eta_{0}-1\right)e^{-\gamma u_{i}}+1$.

Figure 7C shows the reproduction of the reaching skill using the divergent strategy (i.e., η_0_ = 0.55 and η_*f*_ = 1). The reproduction uses the exponential growth to diverge from the directrix toward the boundary of the GC while achieving the main goal of the task which is reaching toward the object. Table 1 summarizes the settings and properties of the discussed reproduction strategies.

**Table 1:** A guideline for setting reproduction strategies for TLGC.

**Strategy**	**Setting**	**Property**
Fixed-ratio	Use (8), (9), (10) with η_*f*_ = η_0_	Preserves shape
Convergence	Use (8), (9), (10) with η_*f*_ = 0	DMP-like reproduction
Divergence	Use (8), (9), (10) with η_*f*_ = 1	Avoids demonstration space
Adaptive	use (8), (9), (10) with η_*f*_ = automatically	Obstacle avoidance

### 4.3. Generalization

The approach described thus far enables a robot to reproduce the skill inside the GC and under similar start and goal states. However, to have a robust and flexible skill model we must ensure it can generalize to novel situations. We use a nonrigid registration technique (Maintz and Viergever, 1998) to achieve this goal. Given a set of points in source geometry (i.e., the environment during the demonstration) and a corresponding set of points in target geometry (i.e., the environment during reproduction), the nonrigid registration technique computes a spatial deformation function. To adapt to the new states of the skill during reproduction, the constructed generalized cylinder uses this deformation function.

Nonrigid registration techniques have been widely used in medical imaging (Maintz and Viergever, 1998), computer vision (Belongie et al., 2002), and 3D modeling communities (Pauly et al., 2005). Recently, Schulman et al. (2016) demonstrated the usefulness of nonrigid registration in LfD by employing it for autonomous knot tying. Their proposed *trajectory transfer* method is based on the classic Thin Plate Splines (TPS) registration algorithm (Bookstein, 1989) extended to 3D Cartesian space, which we also utilize here.

Consider a source geometry composed of a set of *N* landmark points in 3D Cartesian space, $L=\left\{L_{n}\in {\mathbb{R}}^{3}|n=1,2,\ldots ,N\right\}$, and a target geometry composed of the corresponding set of landmark points, $L^{\prime }=\left\{L_{n}^{\prime }\in {\mathbb{R}}^{3}|n=1,2,\ldots ,N\right\}$. The nonrigid registration problem then is to find an interpolation function **z**:ℝ^3^↦ℝ^3^ constrained to map the points in L to the points in $L^{\prime }$. However, there are infinitely many such interpolation functions. To address this issue, TPS finds an interpolation function that achieves the optimal trade-off between minimizing the distance between the landmarks and minimizing the so-called *bending energy*, in effect finding a smooth interpolator. The TPS formulation is given by

(11) $$\underset{\text{z}}{\min}\left\{\sum_{n=1}^{N}\|{L^{\prime }}_{n}-z(L_{n}){\|}^{2}+\lambda \int_{{\mathbb{R}}^{3}}dx\sum_{i\in \{1,2,3\}}\|{▽}^{2}z_{i}(x){\|}_{F}^{2}\right\}$$

where ${▽}^{2}z_{i}$ represents the Hessian matrix of the *i*th dimension of the image of **z**, λ is a regularization parameter, and ||.||_*F*_ is the Frobenius norm. The integral term represents the bending energy. Minimizing the bending energy term in our case is equivalent to minimizing the dissimilarity between the initial and deformed generalized cylinder (i.e., preserving the shape of the skill). The interpolation function **z** which solves (11) consists of two parts: an affine part and a non-affine part. The affine part approximates the overall deformation of the geometry acting globally, while the non-affine part represents the local residual adjustments forced by individual landmark points. With the non-affine part expanded in terms of the basis function ϕ, **z** can be represented as $\text{z}\left(\text{x}\right)=\text{b}+\text{A}\text{x}+\overset{N}{\underset{n=1}{\sum }}{\text{w}}_{n}\phi \left(L_{n},\text{x}\right)$ where **b** ∈ ℝ^3^, **A** ∈ ℝ^3 × 3^ and ${\text{w}}_{n}\in {\mathbb{R}}^{3}$ are the unknown parameters while the basis function is defined as $\phi \left(L_{n},\text{x}\right)=||L_{n}-\text{x}||\forall \text{x}\in {\mathbb{R}}^{3}$. The unknown parameters **b**, **A**, and **w**_*n*_ can be found using matrix manipulation (Bookstein, 1989).

In the generalization procedure using TPS (detailed in Algorithm 3), the source geometry is composed of the locations of the important landmarks in the workspace during the demonstration while the corresponding target geometry is composed of the new locations of the landmark points. For instance, in the reaching skill, the location of the object is considered as the source landmark and the target landmark is the new location of the object during the reproduction. Given the landmarks, the algorithm first finds the interpolation function **z** using the nonrigid registration method (line 4 in Algorithm 3). The algorithm then uses **z** to transform the directrix **m**↦**m**′ and the cross-sectional function $P\mapsto P^{\prime }$ (lines 5 and 6 in Algorithm 3). The new generalized cylinder $G_{u,v}^{\prime }$ is then constructed using the mapped parameters (line 7 in Algorithm 3). To reproduce the skill in $G_{u,v}^{\prime }$, the reproduction methods from section 4.2 can be employed. It has to be noted that the set of landmarks is not limited to only the initial and final points on the trajectory (e.g., location of the object) and can include any point on the trajectory. In section 7, we use this concept for obstacle avoidance.

**Table:** **Algorithm 3** Generalization of GC using TPS

1:	procedure GeneralizeGC
2:	Input:$G_{u,v}$, frames $F_{u}$, source & target landmarks L, $L^{\prime }$
3:	Output:target $G_{u,v}^{\prime }$, target frames $F_{u}^{\prime }$
4:	$\text{z}\leftarrow \text{findTPS}\left(L,L^{\prime }\right)$*solve* (11)
5:	**m**′(*u*)←**zm**(*u*)
6:	$P^{\prime }\left(u,v\right)\leftarrow \text{z}P\left(u,v\right)$
7:	$G_{u,v}^{\prime },F^{\prime }\leftarrow \text{makeGeneralizedCylinder}\left({\text{m}}^{\prime }\left(u\right),P^{\prime }\left(u,v\right)\right)$

To evaluate the proposed approach, we used TPS to generalize the GC learned for a pick-and-place skill (experiment five in section 5.1) over four novel final states of the task. We selected two landmarks including the locations of the object and the box. While the location of landmark on the object remains unchanged, the location of the target landmark on the box was changed during the reproduction. The result of employing Algorithm 3 are depicted in Figure 10A. It can be seen that the skill can be successfully generalized to the four desired novel locations of the box.

One of the drawbacks of the non-rigid registration technique is that it can lead to non-linear deformations (see section 5.2 for a discussion about the robustness of the TPS approach). To address this issue, alternate generalization techniques such as Laplacian Trajectory Editing (LTE) (Nierhoff et al., 2016) can be used. LTE interprets a trajectory $\text{P}={\left[\text{p}\left(t_{1}\right),\ldots ,\text{p}\left(t_{n}\right)\right]}^{⊤}$ as an undirected graph and assigns uniform umbrella weights, *w*_*ij*_ = 1, to the edges *e*_*ij*_ if *i* and *j* are neighbors and *w*_*ij*_ = 0 otherwise.

Local path properties are specified using the discrete Laplace-Beltrami operator as

(12) $$\begin{array}{r} {\text{δ}}_{i}=\underset{j}{\sum }\frac{w_{ij}}{\sum w_{ij}}\left({\text{p}}_{i}-{\text{p}}_{j}\right). \end{array}$$

For the whole graph, (12) can be written as **Δ** = **LP** where $\text{Δ}={\left[{\text{δ}}_{1},\ldots ,{\text{δ}}_{n}\right]}^{⊤}$ and **L** is defined as

(13) $$L_{ij}=\{\begin{array}{cc} 1 & i=j\\ -w_{ij}/\sum_{j}w_{ij} & \text{isNeighbor}(i,j)\\ 0 & \text{otherwise} \end{array}$$

LTE solves **Δ** = ***LP*** using least square by specifying additional constraints **C** (similar to landmarks in non-rigid registration) as

(14) $$P_{d}={\left[\begin{array}{c} L\\ P \end{array}\right]}^{†}\left[\begin{array}{c} \Delta \\ C \end{array}\right],$$

where ***P***_*d*_ is the deformed graph and † denotes the pseudo-inverse. To handle non-linear deformations, LTE calculates elements of the homogeneous transformation that maps the source landmarks **p**_*L*_*S*_, *i*_ to the target landmarks **p**_*L*_*T*_, *i*_ through Singular Value Decomposition by

(15) $$\begin{array}{r} \underset{\text{z}}{\min}\left\{\overset{k}{\underset{n=1}{\sum }}||{\text{p}}_{d,i}-\text{z}{\text{p}}_{s,i}|{|}^{2}\right\}, \end{array}$$

where **z** is the homogeneous transformation including a scalar scaling factor, a rotation matrix, and a translational vector. The generalization procedure for GCs using LTE is detailed in Algorithm 4. Given the source and target landmark sets, we first calculate the Laplacian (Line 4 in Algorithm 4) and then find the mapping **z** by applying least square and then Singular Value Decomposition to deal with non-linear deformations. We then use **z** to map the directrix **m** ↦ **m**′ and the cross-sectional function $P\mapsto P^{\prime }$ (Lines 6 and 7 in Algorithm 4). The new generalized cylinder $G_{u,v}^{\prime }$ is then constructed using the mapped parameters (line 8 in Algorithm 4). The reproduction methods from section 4.2 can be employed to reproduce the skill in $G_{u,v}^{\prime }$.

**Table:** **Algorithm 4** Generalization of GC using LTE

1:	procedure GeneralizeGC-LTE
2:	Input:$G_{u,v}$, frames $F_{u}$, source & target landmarks L, $L^{\prime }$
3:	Output:target $G_{u,v}^{\prime }$, target frames $F_{u}^{\prime }$
4:	[$\text{L},\Delta ]\leftarrow \text{calcLaplacian}\left(\text{m}\left(u\right),L,L^{\prime }\right)$*using* (12), (13)
5:	$\text{z}\leftarrow \text{SVD}\left({\text{p}}_{d},{\text{p}}_{s},L,L^{\prime }\right)$*using* (15)
6:	**m**′(*u*)←**zm**(*u*)
7:	$P^{\prime }\left(u,v\right)\leftarrow \text{z}P\left(u,v\right)$
8:	$G_{u,v}^{\prime },F^{\prime }\leftarrow \text{makeGeneralizedCylinder}\left({\text{m}}^{\prime }\left(u\right),P^{\prime }\left(u,v\right)\right)$

Similar to Algorithm 3, we evaluated Algorithm 4 for the pick-an-place task over the same four final situations. The location of the box and the ball are considered as landmarks. The result of employing Algorithm 4 are depicted in Figure 10C. It can be seen that the skill can be successfully generalized to the four desired novel locations of the box during reproduction and the deformed GCs are very similar to the ones obtained using Algorithm 3. For a discussion about the robustness of the LTE approach compared against TPS see section 5.2.

## 5. Experimental Results

We conducted eight experiments on two robotic platforms to demonstrate the encoding of the GC model, as well as its reproduction and generalization capabilities, on multiple trajectory-based skills^8^. For each experiment, we gathered a set of demonstrations through kinesthetic teaching using either a 6-DOF Kinova Jaco2 robot or a 7-DOF Sawyer robotic arm (Figures 1, 3). The data was recorded at 100 Hz.

### 5.1. Learning and Reproduction

In this section, we present examples of six trajectory-based skills encoded using the generalized cylinder model. In the first experiment, we performed a reaching skill toward an object (green sphere) from above (Figure 5). We present circular, elliptical and closed-spline cross-sections to showcase how GCs with different cross-section types encode the demonstration space. Ten reproductions of the skill from various initial poses produced by the fixed-ratio rule are depicted in Figure 9A.

**Figure 8:**
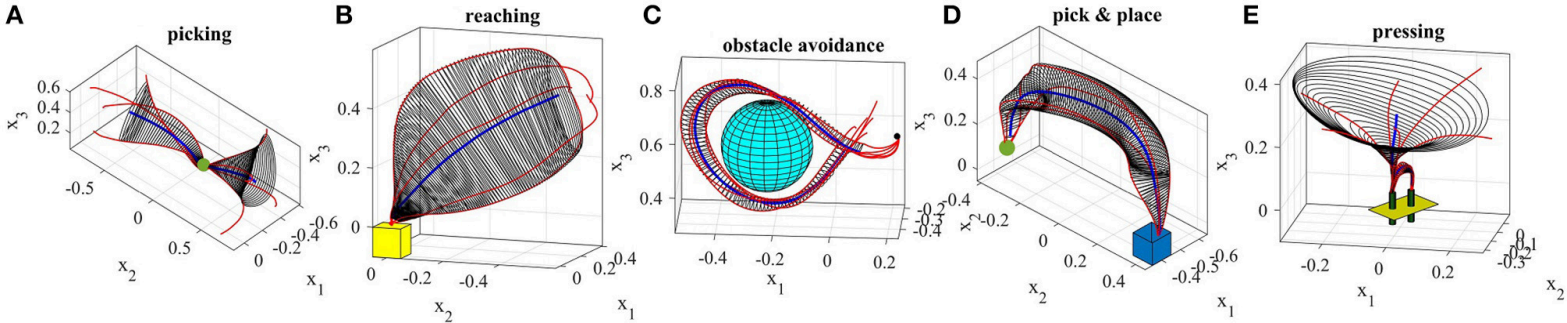
Five real-world experiments performed using TLGC. The skills are encoded by extracting the directrix (blue) and generalized cylinder (gray) from demonstrations (red).

**Figure 9:**
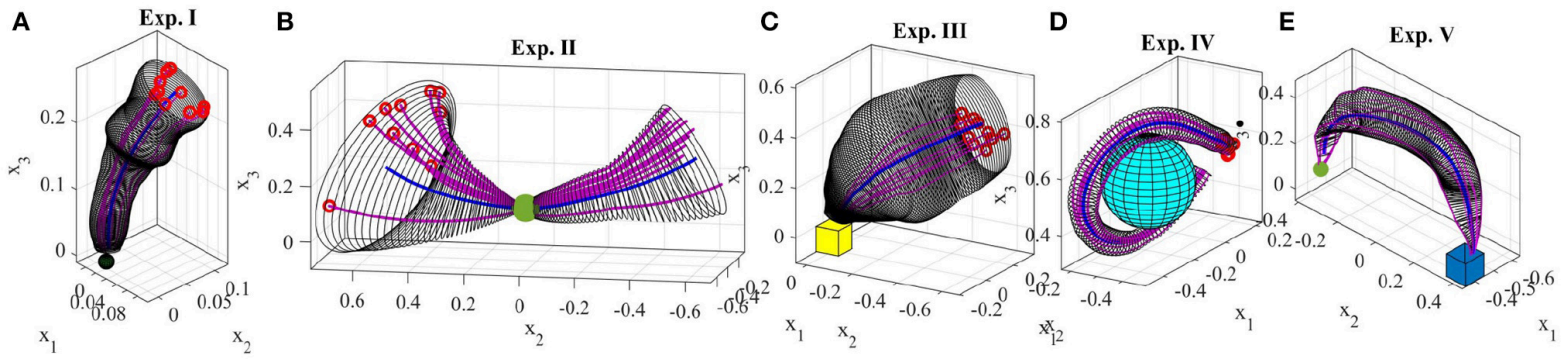
Ten reproductions of the skill for each experiment. The reproduced trajectories (magenta) are generated using the fixed-ratio rule from arbitrary initial poses.

The demonstrations recorded for the second experiment (Figure 8A) show an example of a movement that can be started and ended in a wide task-space but in the middle is constrained to pass through a narrow area. This movement resembles threading a needle or picking up an object in the middle of the movement. The obtained GC extracts and preserves the important characteristics of the demonstrated skill, i.e., the precision and shape of the trajectory throughout the movement. Figure 9B shows 10 successful reproductions of the skill from various initial poses using the fixed-ratio rule. LfD approaches such as SEDS (Khansari-Zadeh and Billard, 2011) that require a single end point fail to model this skill successfully.

**Figure 10:**
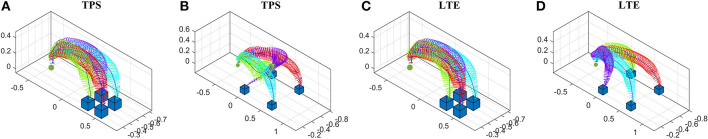
Generalization of the learned pick-and-place skill over four novel final states. **(A,B)** Using TPS through Algorithm 3, and **(C,D)** using LTE through Algorithm 4. New locations of the box in **(A,C)** are close to the original location of the box, while the new box locations are farther in **(B,D)**.

The third experiment (Figure 8B) shows a reaching/placing skill similar to the first experiment with a curved trajectory. The robot learns to exploit a wider demonstration space while reaching the object and maintaining trajectories with precision near the object. Figure 9C illustrates ten reproductions of the skill from various initial poses produced by the fixed-ratio rule^9^.

The fourth experiment (Figure 8C) shows a circular movement around an obstacle, which is unknown to the robot. Since the given demonstrations avoid the obstacle, and the encoded GC guarantees that all the reproductions of the task remain inside the cylinder, the reproduced path is guaranteed to be collision-free. Figure 9D illustrates ten reproductions of the skill from various initial poses produced by the fixed-ratio rule. It can be seen that all the reproductions stay inside the boundaries while exploiting the demonstration space represented by GC. Figure 1 (middle) also shows a snapshot captured during the reproduction of the skill.

The fifth task represents a pick-and-place movement in which the robot picks up an object and places it in a box (Figure 8D). The encoded GC shows that the initial and final poses of the movement are the main constraints of the task while in the middle of the trajectory, the end-effector can pass through a wider space while preserving the shape of the movement. Figure 1 (left) shows a snapshot during the reproduction of this skill. Figure 9E depicts ten reproductions of the skill generated by the fixed-ratio rule.

The sixth experiment illustrates a pressing skill with multiple goals [Figure 1(right)]. The robot starts from a wide demonstration space, reaches to the first peg, presses it down, retracts from it, reaches for the second peg, and presses it down (Figure 8E). Unlike many existing LfD approaches, TLGC can handle this skill although it includes more than one goal and does not require skill segmentation. In addition, to show that the proposed approach is robot-agnostic, we have conducted this experiment on a 7-DOF Sawyer robot.

### 5.2. Generalization

In this section, we demonstrate the generalization capability of the proposed approach using data from the fifth experiment. By detecting a change in the location of the objects during the reproduction the generalization is performed to satisfy the new conditions. After encoding the skill in the fifth experiment (Figure 8D), we relocated the box four times and each time used Algorithm 3 to adapt the encoded model to the new situations. The results can be seen in Figure 10A. We then repeated the experiment by employing Algorithm 4. Figure 10C illustrates the results. As noticeable in both generalization experiments, the overall shape of the generalized cylinders is preserved while accordingly expanding or contracting for different final poses. It also can be seen that the two experiments resulted in very similar GCs.

As mentioned before, one of the drawbacks of the non-rigid registration technique is that it leads to non-linear deformations as the distance between the source and target landmark locations increases. Figure 10B shows an example of such instability caused by increasing the distance of the target landmark (i.e., the box) from the source landmark (i.e., initial location of the box). Although the magenta GC in Figure 10B satisfies the initial and final states of the task in the new environment, it does not preserve the shape of the skill. On the other hand, when employing Algorithm 4 for the same source and target landmark locations, the results depicted in Figure 10D indicate that LTE is more robust to the increase in the distance between the source and target landmarks. Unlike TPS, the magenta GC generalized using LTE not only satisfies the initial and final states of the skill but also preserves the shape of the skill as close as possible. As a direct consequence of the ratio rule for reproduction, this successfully enables reproduction of the skill to unforeseen situations while preserving the important features of the skill.

### 5.3. Comparison to DMPs and GMM/GMR

In this section, we compare the presented approach to two widely-used LfD techniques, Dynamic Movement Primitives (DMPs) (Ijspeert et al., 2013), and Gaussian Mixture Model/Gaussian Mixture Regression (GMM/GMR) (Calinon et al., 2007). Since the original DMP representation (Ijspeert et al., 2013) is limited to learn from a single demonstration, we compare our approach to a variant of DMPs that constructs a representation from a set of demonstrations (Calinon et al., 2010). We performed two experiments comparing the behavior of the above approaches to TLGC.

#### 5.3.1. Comparison I

**Figure 11:**
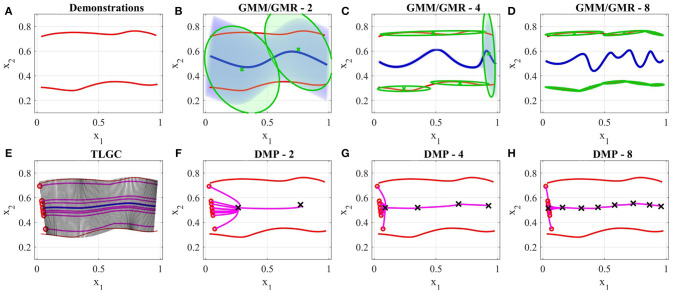
Results of the comparison between GMM, DMPs, and TLGC in section 5.3.1 **(A)** demonstrations **(B–D)** GMM with two, four, and eight components respectively, **(E)** generalized cylinder and ten reproductions **(F–H)** DMPs with two, four, and eight attractors and 10 reproductions, respectively.

Figure 11A shows two demonstrations of a skill performed by a teacher. The demonstrations are simple direct trajectories (i.e., movement of robot's end-effector from left to right). Unlike many common scenarios, in this case the size of the demonstration space does not decrease at the end of the skill. When providing such demonstrations, the user is showing not only the shape of the movement but also the boundaries of the movement. The goal of this experiment is to show that existing approaches have not been designed to deal with the demonstration space and this fact serves as one of the main motivations for proposing TLGC.

We first employed TLGC with a circular cross-section and generated 10 reproductions from various initial poses (Figure 11E). The encoded GC represents the demonstration space and the reproductions maintain the important characteristics of the movement. TLGC requires no parameter tuning and can reproduce multiple nonidentical yet valid solutions in the demonstration space. For GMM/GMR we tuned the number of Gaussian components to 2, 4, and 8, in Figures 11B–D, respectively, and for DMPs, we tuned the number of attractors to 2, 4, and 8, in Figures 11F–H, respectively. The results show that both DMPs and GMM/GMR are capable of learning the skill. However, unlike TLGC, GMM/GMR reproduces a single solution for this task. The reproduced trajectory by GMM/GMR imitates the demonstrations, however, the trajectory oscillates in the middle under the influence of different Gaussian components placed at the demonstration space. As shown in Figures 11C,D, increasing the number of components to four and then to eight fails to improve the shape of the reproduced trajectory and although the amplitude decreases the frequency increases. For DMPs, increasing the number of attractors helps with imitating the given demonstrations. However, starting from different initial points, reproduced trajectories converge to an average solution (similar to the directrix in TLGC). This experiment highlights the role and the importance of the demonstration space on the behavior of the learning approach.

#### 5.3.2. Comparison II

**Figure 12:**
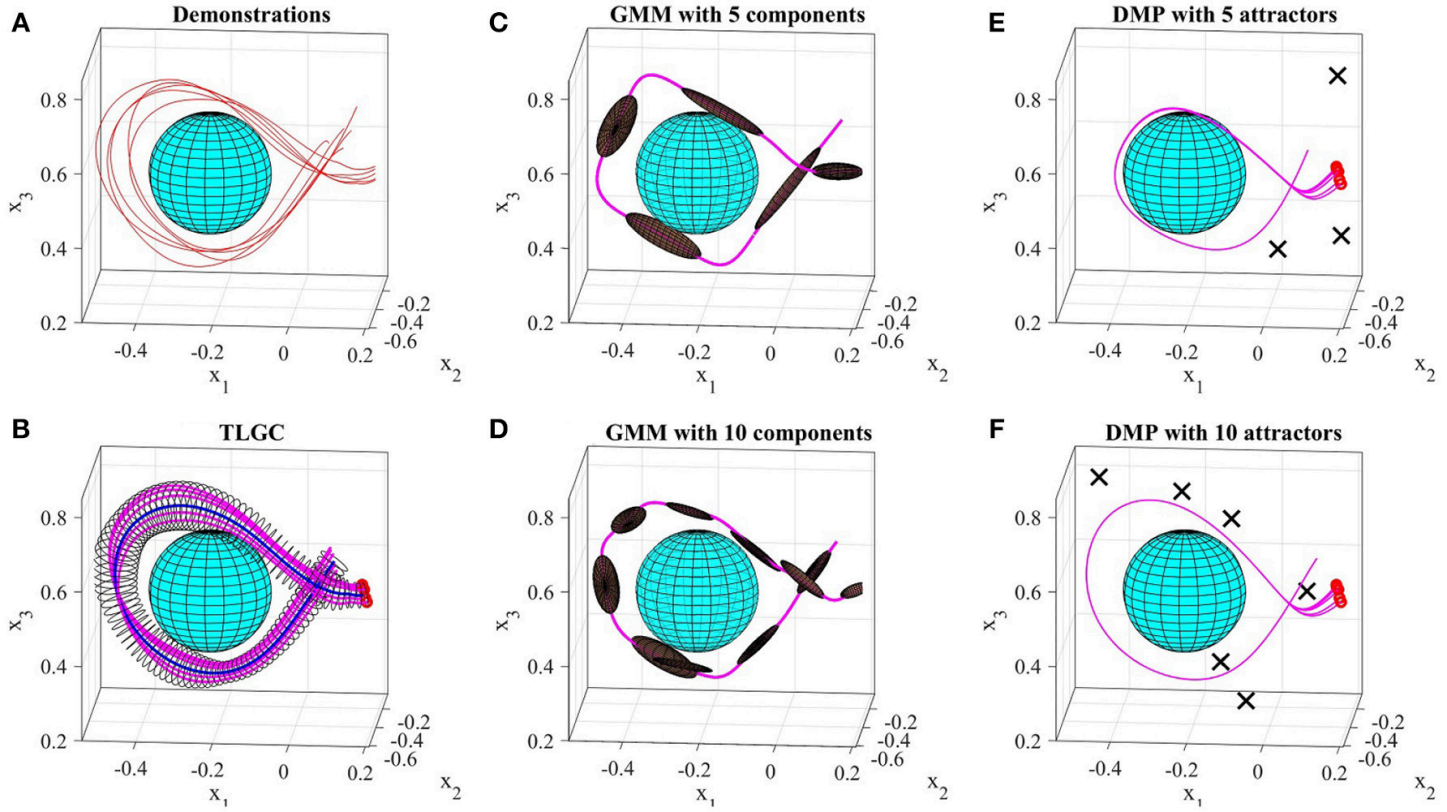
Results of the comparison between GMM, DMPs, and TLGC in section 5.3.2 **(A)** demonstrations **(B)** GC and five reproductions, **(C,D)** GMM with five and ten components, respectively, **(E,F)** DMPs with five and ten attractors, respectively and five reproductions.

Figure 12 shows a comparison of results on the data from experiment four. The demonstrations and the object are shown in Figure 12A. For TLGC, we encoded the demonstrations using a generalized cylinder with a closed-spline cross-section and generated five reproductions from various initial poses (Figure 12B). As mentioned before, TLGC requires no parameter tuning beyond specifying the cross-section type, and by extracting the characteristics of the movement it learns to avoid the obstacle. For GMM/GMR we tuned the number of Gaussian components to 5 and 10, in Figures 12C,D, respectively, and for DMPs, we tuned the number of attractors to 5 and 10, in Figures 12E,F, respectively. The results show that both DMPs and GMM/GMR can learn the skill. In Figure 12C, and to a lesser degree in Figure 12D, GMM/GMR produces a more angular trajectory than seen in the demonstrations. In Figure 12E we see that DMPs deviating from the demonstrations and colliding with the object. In all four examples, we also observe that the five reproductions starting from different initial locations converge to a single path. In contrast, the reproductions by TLGC produce a more natural set of motions that are not identical and exploit the demonstration space while preserving the shape of the skill. Note that TLGC can reproduce analogous trajectories by using the convergent reproduction strategy if that behavior is desirable.

## 6. Skill Refinement

So far, we have shown that TLGC can be used to extract, reproduce, and generalize skills from *reliable* human demonstrations. In practice, however, due to morphological differences between the robot and the human, user-provided demonstrations of a task are usually *sub-optimal*. Multiple solutions to address this problem have been proposed. Argall et al. (2009a) showed that a skill can be corrected by having the teacher to assign weights to each demonstration based on its quality. However, assigning weights to demonstrations is not trivial. Another solution is to start from the sub-optimal model and explore better solutions. For instance, an LfD approach can be combined with Reinforcement Learning to refine the sub-optimal behavior of the model (Kormushev et al., 2010). This family of solutions usually suffer from two drawbacks (a) a manually engineered reward function is required, and (b) finding an improved solution entails extensive trial and error. An alternative approach is to refine the skill through physical human-robot interaction (Argall et al., 2010). In this work, we differentiate two types of refinement. *Incremental Refinement* occurs during the learning process in which the user applies modifications while the robot is replaying a demonstration (or a reproduction) and the updated data is used to retrain the model. Once a model is learned, *constraint-based refinement* can be used to refine the model further by applying new constraints. In this section, we show that both approaches can be applied to TLGC. Note that we have selected simple tasks for analysis and illustrative purposes.

### 6.1. Incremental Refinement

In its first form, skill refinement can be performed during the learning process incrementally. After encoding the skill using TLGC, the user identifies a *target trajectory* (either a demonstration or a reproduction) that needs to be modified. We execute the target trajectory with the robot in compliant control mode, allowing the joints and the end-effector position to be adjusted while the robot is moving. Therefore, while the robot is replaying the target trajectory, the teacher can reshape the movement through kinesthetic correction. The obtained trajectory either replaces the initial demonstration or is added to the set as a new demonstration. Given the new set, the algorithm updates the model and reproduces new trajectories that inherit the applied corrections.

To evaluate this method, we initially demonstrated three simple trajectories and encoded the skill with a closed-spline cross-section (**Figure 14A**), and reproduced the skill using the fixed-ratio rule (**Figure 14B**). Now assume we would like the robot end-effector to dip downwards in the middle of the first (top) demonstration. While the robot is replaying the target demonstration, the teacher reshapes the demonstration through kinesthetic correction. **Figure 14C** illustrates the original and refined demonstrations. **Figure 14D** shows the updated GC after replacing the target with the refined demonstration, as well as a reproduction of the skill from a given initial point, that reflects the performed refinements^9^. This experiment shows that TLGC can be used to refine a learned skill incrementally. Although many approaches can benefit from a similar process (Argall et al., 2010), our representation is visually perceivable and has the potential to enable even non-experts to observe and interpret the effects of the refinement on the model.

### 6.2. Constraint-Based Refinement

**Figure 13:**
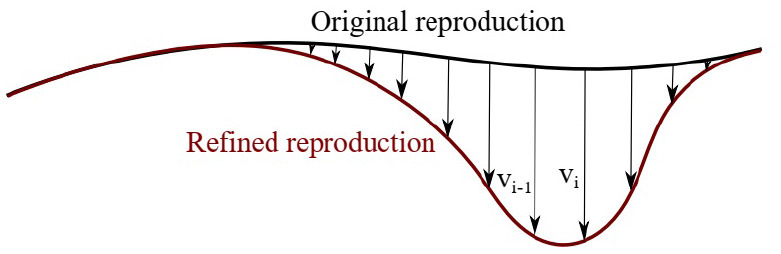
Formation of the refinement matrix from the original and modified reproductions.

In this section, we show that skill refinement can be performed after the model has been encoded by applying new constraints. We consider the skill from previous experiment (**Figure 15A**). Assume during a reproduction, the user observes and kinesthetically modifies the reproduced trajectory. When a correction is imposed, we compare the original and the modified trajectories, calculate point-to-point translation vectors **v**_*i*_, and form a refinement matrix $\hat{V}$, by concatenating the vectors. Figure 13 depicts the formation of the refinement matrix. The refinement matrix acts as a geometric constraint on the GC that would affect future reproductions. The green trajectory in **Figure 15C** shows how the reproduction in **Figure 15B** is refined by the teacher through kinesthetic correction; the teacher has applied downward forces (−*x*_3_ direction) to keep the end-effector at a certain level. We calculated the refinement matrix $\hat{V}=\left[{\text{v}}_{1},\ldots ,{\text{v}}_{n}\right]\in {\mathbb{R}}^{3\times n}$ and applied it as a constraint to our fixed-ratio rule. A reproduction remains unaffected if it is generated below the constraining plane. This case can be seen as the lower reproduction in **Figure 5D**. On the other hand, if a reproduction intersects with the constraining plane, the refinement matrix applies to it. The upper reproduction in **Figure 15D** shows the effect of the constraint while the dashed line shows reproduction without applying the constraint^9^. This experiment indicates that using constraint-based refinement, the user can apply new constraints to the model without modifying it. One of the advantages of this approach is that the imposed constraints later can be removed or combined with other constraints without updating the encoded model. To our knowledge there is no other LfD approach with similar capabilities.

**Figure 14:**
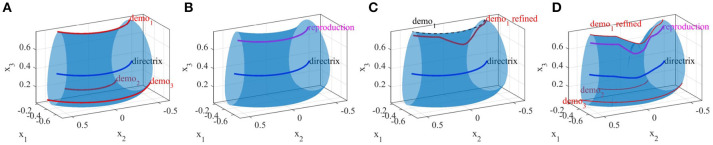
Incremental refinement of a skill by correcting a demonstration. **(A)** Demonstrations (red), directrix (blue) and the obtained GC, **(B)** reproduction from a random pose (magenta), **(C)** first demonstration was refined (red) by user, **(D)** updated GC, directrix, and a new reproduction.

**Figure 15:**
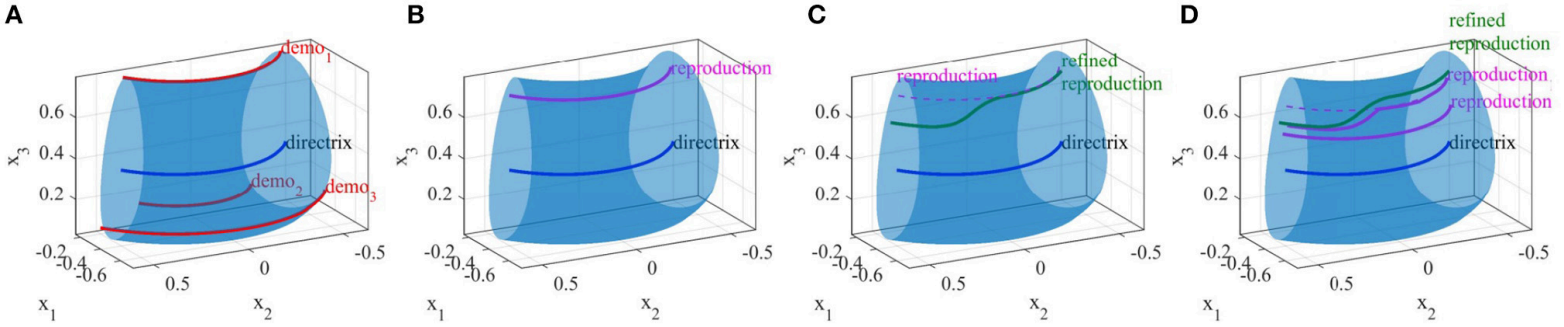
Constraint-based refinement of a skill by correcting a reproduction. **(A)** Demonstrations (red), directrix (blue), and the obtained GC, **(B)** reproduction from a random pose (magenta), **(C)** refined reproduction (green), **(D)** two new reproductions; upper one affected by the refinement, while lower one is not.

Note that, the refined reproduction might not be continuous at the point where the constraint applies first (see the top reproduction in Figure 15D). However, our experiments show that the low-level controller of the robot can handle this during execution^9^. Another solution is to smooth the trajectory before execution.

### 6.3. Comparison to GMM-wEM

As mentioned before, the approach proposed by Argall et al. (2010) enables a human to refine the trajectories during the learning process using tactile feedback. The refined trajectories are later used as new demonstrations to reproduce the skill through incremental learning. Their approach GMM-wEM combines GMM/GMR with a modified version of the Expectation-Maximization algorithm that uses a forgetting factor to assign weight values to the corrected points in the dataset. In this section, we compare TLGC to GMM-wEM on two refinement experiments.

#### 6.3.1. Comparison I

**Figure 16:**
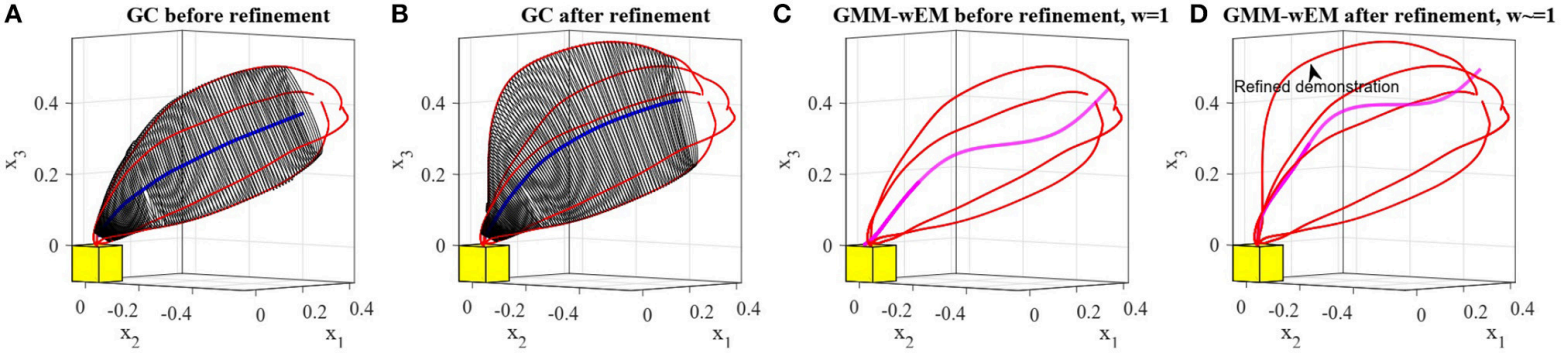
Comparing incremental skill refinement on the reaching skill in section 5.1. **(A,B)** results using TLGC, **(C,D)** results using GMM-wEM.

In the first comparison, we repeated the third experiment from section 5.1, refined one of the four demonstrations, replaced it in the set, and retrained the model. We performed this experiment using both TLGC and GMM-wEM as presented in Argall et al. (2010). Figures 16A,B depict that the model encoded using TLGC adapts to the new set and updates the demonstration space. It can be seen that the directrix is also moved toward the new demonstration. Figures 16C,D show the results for GMM-wEM where the encoded Gaussian model with three components also adapts to the new set of demonstrations. However, the reproduction, both before and after the refinement, oscillates toward different Gaussian components. Although the reproduction achieves the goal of the task, it is dissimilar to the demonstrations.

#### 6.3.2. Comparison II

**Figure 17:**
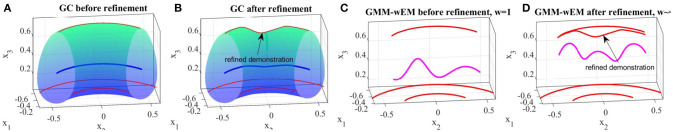
Comparing incremental skill refinement on the first experiment in section 6.1. **(A,B)** results using TLGC, **(C,D)** results using GMM-wEM.

In the second comparison, we repeated the refinement experiment in section 6.1. Since the refined demonstration replaces the original trajectory in the set, for GMM-wEM we assign weight values 0 and 1 to the original and refined demonstrations respectively. As depicted in Figures 17A,B, given the refined trajectory and using TLGC, the encoded model is adapted to the new set and can reproduce new trajectories accordingly. However, as illustrated in Figures 17C,D, because of the wide demonstration space, the model encoded using GMM-wEM cannot represent the skill properly and the reproduction of the skill is oscillating toward different Gaussian components. The reproduced trajectories using the updated GC exploit the whole demonstration space while maintaining the main and refined characteristics of the skill. GMM-wEM, on the other hand, fails to represent the demonstration space.

## 7. Obstacle Avoidance

In this section, we discuss a few strategies for dealing with stationary obstacles using TLGC. Handling dynamic obstacles is out of the scope of this article. A stationary obstacle can either be known and present during both the demonstration and the reproduction phases or just appear during the reproduction phase. We refer to the first case as *known* obstacles and the second case as *unknown* obstacles. We discuss each scenario separately in the following sections.

### 7.1. Avoiding Known Obstacles

For a known stationary obstacle which is present during both the demonstration and the reproduction, we can safely assume that it was avoided by the teacher during the demonstration phase. This means that neither the set of demonstrations nor the formed demonstration space intersects with the obstacle. As we have shown in experiment four (Figure 8C), the constructed GC with convex cross-sections avoids the obstacle. Therefore, the reproduced trajectories from this GC also will avoid the obstacle as long as the obstacle remains stationary. While this feature is inherent to our representation, the process becomes more complicated when the obstacle is not present during the demonstration phase. Note that, a GC with circular cross-sections that encodes the skill might intersect with the obstacle, since it can bound an unintended volume as the demonstration space (Figure 4).

### 7.2. Avoiding Unknown Obstacles

It should be noted that the term *unknown* obstacle refers to the scenario where a stationary obstacle is not present during the demonstration but can be detected during the reproduction. We propose two methods for avoiding unknown obstacles that intersect with the constructed generalized cylinder.

#### 7.2.1. Method I

**Figure 18:**
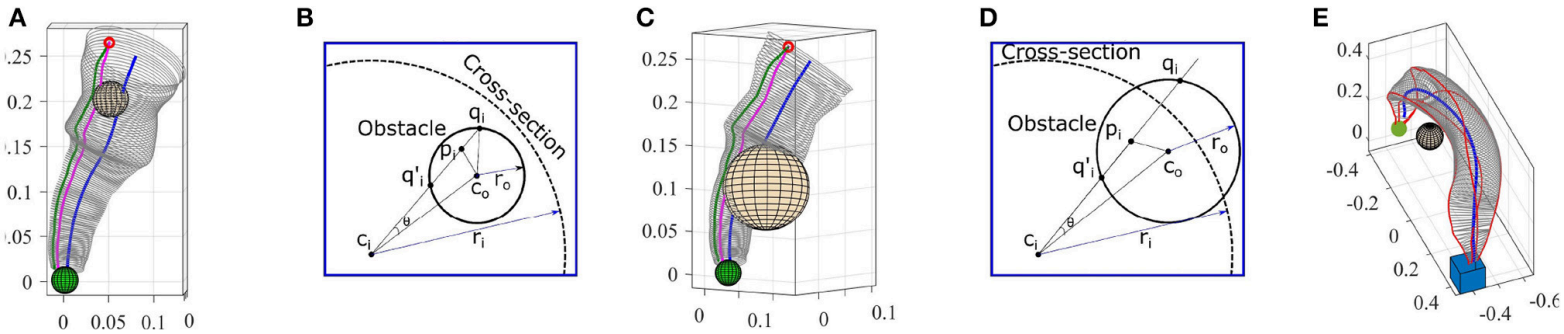
**(A,C)** Reproductions using fixed-ratio strategy (magenta) collide with obstacles. Adapted reproductions using adaptive-ratio strategy (green) avoid obstacles. **(B,D)** Planar views for calculating final ratio. **(E)** Pick-and-place in the presence of an obstacle. GC was deformed using Algorithm 4 to avoid collision.

Consider the GC constructed for the reaching task in the first experiment. Now we assume an obstacle was detected during the reproduction. In the first step, we estimate a bounding sphere for the detected obstacle. Among several existing algorithms, we use Fischer's algorithm (Fischer et al., 2003) which is fast and efficient. Geometrically, an obstacle can either be inside the GC or partially intersect with it. Both cases are illustrated in Figures 18A,C. We intentionally select an identical initial pose for which in both cases reproducing the skill using the fixed-ratio rule (with ratio η_0_) causes collision with the obstacles (magenta trajectories in Figures 18A,C).

Our goal is to generate a trajectory that adapts to the new condition and avoids the obstacle while preserving the main characteristics of the skill as much as possible. Given the size and location of the obstacle (i.e., the bounding sphere), our method calculates a new ratio η_*f*_ and a decay constant γ accordingly and employs the adaptive-ratio rule (section 4.2.1) to generate a collision-free reproduction of the skill.

We use the diagrams depicted in Figures 18B,D to explain our method for estimating the final ratio η_*f*_ in both cases. Assume the sphere representing the obstacle is centered at point *c*_*o*_ with radius *r*_*o*_. We first find *c*_*i*_ the closest point on the directrix to the center of the sphere *c*_*o*_. We use the corresponding cross-section of the GC centered at *c*_*i*_ for our calculation. In the next step, we find *p*_*i*_ the closest point on the reproduced trajectory with the fixed-ratio rule to *c*_*o*_. The cosine of θ which is the angle between $\bar{c_{i}c_{o}}$ and $\bar{c_{i}p_{i}}$ can be calculated as $\cos\theta =\left(\bar{c_{i}c_{o}}\right).\left(\bar{c_{i}p_{i}}\right)/\left(\left||\bar{c_{i}c_{o}}||||\bar{c_{i}p_{i}}|\right|\right)$. Since $||\bar{c_{o}q_{i}}||=r_{o}$, we can find the points where $\bar{c_{i}p_{i}}$ intersects with the obstacle by solving the quadratic equation given by $x^{2}-2x\left(\bar{c_{i}c_{o}}\right)\cos\theta +\left({\left(\bar{c_{i}c_{o}}\right)}^{2}-{\left(r_{o}\right)}^{2}\right)=0$ for △(*c*_*o*_*q*_*i*_*c*_*i*_). From the set of solutions $x=\left\{q_{i},q_{i}^{\prime }\right\}$, we consider ones that are inside the generalized cylinder (the solutions that satisfy $\bar{xc_{i}}\leq r_{i}$). Both solutions in Figure 18B are valid while in Figure 18D, *q*_*i*_ is outside the GC and hence invalid. For each valid solution, we can calculate a final ratio that forces the reproduction to pass through that point. For instance, in Figure 18B, the final ratio for passing through *q*_*i*_ can be calculated as $\eta_{f}=||\bar{c_{i}q_{i}}||/r_{i}$.

Now we estimate the decay constant γ that defines how fast the final ratio η_*f*_ is reached. This constant can be estimated by finding *u*_*c*_ the minimum distance from the initial pose *p*_0_ to the sphere along the directrix. The ratio at arc-length *u*_*c*_ should reach and stay within a range of certain percentage^10^ of η_*f*_. We call this the critical ratio denoted by η_*c*_. Finally, we can calculate the critical constant decay γ_*c*_ by substituting η_*c*_ and *u*_*c*_ in (10) and solving for γ that gives γ_*c*_ = (1/*u*_*c*_)ln((η_*o*_−η_*f*_)/(η_*c*_−η_*f*_)).

By employing the adaptive-ratio strategy with the calculated η_*f*_ and γ_*c*_, we see in Figures 18A,C that the adapted reproductions avoid the obstacle. It has to be noted that the reproduced trajectory using our method is tangent to the sphere at the intersection point *q*_*i*_. By increasing the size of the bounding sphere (e.g., increasing the radius by a certain percentage), we can force the reproduction to avoid the obstacle from a safe distance. The proposed method is computationally efficient and requires no parameter tuning. As soon as an obstacle is detected the method automatically generates an adapted reproduction of the skill. This method also can be easily extended to GCs with closed-spline cross-sections. One of the limitations of this approach, however, is the assumption of spherical obstacles which in some cases can be inefficient and perform an unnecessary deformation. For instance, when dealing with cylindrical or cubical obstacles.

#### 7.2.2. Method II

Alternatively, a collision can be avoided by deforming the GC using our generalization methods in section 4.3. In practice, this deformation strategy behaves similarly to path planners when dealing with obstacles. However, in skill learning, since the shape of the movement is important, the dissimilarity between the original and the deformed GCs should be minimized. To achieve this, similar to the previous method, we first estimate a bounding sphere for the detected obstacle. Nierhoff et al. (2016) have shown that by considering an obstacle as a positional constraint in (14), the trajectory can adapt to avoid the obstacle. Using this feature and by introducing the bounding sphere as a positional constraint in Algorithm 4, we estimate a transformation function that deforms the GC to avoid the obstacle satisfying the landmarks and preserving the shape of the movement as much as possible. We have evaluated this modification to our generalization algorithm for the task of pick-and-place in experiment five. The deformed GC shown in Figure 18E avoids the obstacle and can be used to generate collision-free trajectories. Since representing non-spherical obstacles as positional constrains is nontrivial, this method also might deform the GC more than required when dealing with non-spherical obstacles.

## 8. Conclusions

We have presented a novel LfD approach for learning and reproducing trajectory-based skills using a geometric representation which maintains the crucial characteristics and implicit boundaries of the skill and generalizes it over the initial and final states of the movement. The proposed approach, TLGC, represents and exploits the demonstration space to reproduce a variety of successful movements. TLGC requires minimal parameter tuning that not-only simplifies the usage of the algorithm and makes the result consistent but also can make the approach more convenient for non-expert users. We have shown that TLGC enables users to refine a learned skill through both incremental and constraint-based refinement strategies interactively. We also have introduced three obstacle avoidance strategies for TLGC and have compared it to two existing LfD approaches.

## Author Contributions

SA and SC significantly contributed to the development of the presented approach, execution of the experiments, analysis of the results, and preparation of the manuscript. Authors approved the submitted version of the manuscript.

### Conflict of Interest Statement

The authors declare that the research was conducted in the absence of any commercial or financial relationships that could be construed as a potential conflict of interest.

## Footnotes

^1^This work focuses on LfD approaches that encode trajectory-based skills (i.e., movements) and we do not discuss goal-based LfD approaches.

^2^A plane simple closed-curve, also known as the Jordan curve, is a continuous loop that divides the plane into an interior region and an exterior region.

^3^A regular curve is a differentiable curve whose derivatives never vanish.

^4^A pencil is a family of geometric objects sharing a common property (e.g., spheres).

^5^An implicit surface is a surface in Euclidean space that can be represented as a function *F* defined by equation *F*(*x*_1_(*u*), *x*_2_(*u*), *x*_3_(*u*)) = 0.

^6^An envelope is a curve/surface tangent to a family of curves/surfaces (2D or 3D).

^7^https://github.com/rezaahmadzadeh/TLGC

^8^In all of the figures, the demonstrations, directrix, and reproductions are plotted in red, blue, and magenta, respectively.

^9^The accompanying video shows the execution of the task https://youtu.be/KqUgT72G8Pw

^10^We empirically found 2−5% is a good range.
